# LKR/SDH Plays Important Roles throughout the Tick Life Cycle Including a Long Starvation Period

**DOI:** 10.1371/journal.pone.0007136

**Published:** 2009-09-23

**Authors:** Banzragch Battur, Damdinsuren Boldbaatar, Rika Umemiya-Shirafuji, Min Liao, Badgar Battsetseg, DeMar Taylor, Badarch Baymbaa, Kozo Fujisaki

**Affiliations:** 1 Laboratory of Emerging Infectious Diseases, Department of Frontier Veterinary Medicine, Faculty of Agriculture, Kagoshima University, Korimoto, Kagoshima, Japan; 2 Institute of Veterinary Medicine, Mongolian State University of Agriculture, Zaisan, Ulaanbaatar, Mongolia; 3 Graduate School of Life and Environmental Sciences, Tsukuba University, Tennohdai, Tsukuba, Ibaraki, Japan; Institut Pasteur, France

## Abstract

**Background:**

Lysine-ketoglutarate reductase/saccharopine dehydrogenase (LKR/SDH) is a bifunctional enzyme catalyzing the first two steps of lysine catabolism in plants and mammals. However, to date, the properties of the lysine degradation pathway and biological functions of LKR/SDH have been very little described in arthropods such as ticks.

**Methodology/Principal Findings:**

We isolated and characterized the gene encoding lysine-ketoglutarate reductase (LKR, EC 1.5.1.8) and saccharopine dehydrogenase (SDH, EC 1.5.1.9) from a tick, *Haemaphysalis longicornis*, cDNA library that encodes a bifunctional polypeptide bearing domains similar to the plant and mammalian LKR/SDH enzymes. Expression of LKR/SDH was detected in all developmental stages, indicating an important role throughout the tick life cycle, including a long period of starvation after detachment from the host. The LKR/SDH mRNA transcripts were more abundant in unfed and starved ticks than in fed and engorged ticks, suggesting that tick LKR/SDH are important for the starved tick. Gene silencing of LKR/SDH by RNAi indicated that the tick LKR/SDH plays an integral role in the osmotic regulation of water balance and development of eggs in ovary of engorged females.

**Conclusions/Significance:**

Transcription analysis and gene silencing of LKR/SDH indicated that tick LKR/SDH enzyme plays not only important roles in egg production, reproduction and development of the tick, but also in carbon, nitrogen and water balance, crucial physiological processes for the survival of ticks. This is the first report on the role of LKR/SDH in osmotic regulation in animals including vertebrate and arthropods.

## Introduction

Amino acids are required for protein biosynthesis, but also have additional functions such as nitrogen storage and transport. Deficiencies or excesses of one or more essential amino acids limit protein synthesis and growth [1]. Lysine (Lys) is an essential amino acid and is required during embryonic development [2], and plays important physiological roles in both plants and animals. Although not fully elucidated, Lys catabolism apparently serves additional physiological and developmental functions in plants and animals [3]–[5]. The first enzyme in the Lys catabolic pathway, Lys-ketoglutarate reductase (LKR), combines Lys and α-ketoglutarate to form saccharopine. Saccharopine is subsequently oxidized by the saccharopine dehydrogenase (SDH) to α-amino adipic semialdehyde and glutamate. α-Amino adipic semialdehyde is degraded to α-aminoadaipate further converted to acetyl-CoA by several enzymatic reactions or alternatively used to produce pipecolic acid [6]. The LKR and SDH enzymes play a pivotal role in Lys catabolism and are linked on a single bifunctional LKR/SDH polypeptide encoded by a single LKR/SDH gene in plants and animals [6]. Lysine catabolic pathway can promptly respond to and influence both the nitrogen and carbon balances in the organism. Under regular growth conditions, expression of the LKR/SDH gene is strongly up-regulated by lysine in floral organs and developing seeds [7], also suggesting an important role in plant reproduction. Moreover, because one of the end products of Lys catabolism is acetyl-CoA, Lys catabolism likely participates in the regulation of carbon/nitrogen partitioning, particularly under metabolic starvation and in senescence of plants [7]. The Lys catabolic pathway is an important route for balancing Lys levels, mostly, because high levels of Lys are toxic to organisms [8]. LKR/SDH enzyme activities are important for Lys catabolism in the mammalian liver and kidney [9], [10], contributing not only to the general nitrogen balance but also the controlled conversion of lysine into ketone bodies [9]–[12]. In plants, the LKR level was shown to be significantly up-regulated in inflorescence tissues and developing seeds, as well as in response to osmotic stress [3], [4], [13], [14]. LKR/SDH gene is also up-regulated by osmotic stress in the rapeseed, *Brassica napus*, leaf and LKR/SDH and SDH mRNAs are produced from a single gene [15]. Osmo-regulation by the LKR/SDH system has also been confirmed through changes occurring at the level of both activities in plant material exposed to different environmental conditions [16]. However, to our knowledge, the role of LKR/SDH in osmoregulation has not been reported in animals including vertebrates and arthropods.

Ticks are among the most significant blood-sucking arthropods worldwide. They transmit various pathogens that can cause disease and death in people, domesticated animals and wild animals [17]. The ixodid tick *Haemaphysalis longicornis* is mainly distributed in East Asia and Australia [18], [19], and is a vector of a wide range of pathogens including *Babesia* and *Theileria* (protozoa), *Borrelia* (bacteria) and viruses that cause hemorrhagic fever and encephalomyelitis [20]. Ticks are obligate haematophagous ectoparasites. All three-life stages (larval, nymphal and adult) are obligate blood feeders with long non-parasitic periods of free-living between blood meals. For example, it is estimated that the sheep tick, *Ixodes ricinus*, spends 97% of its 2–5 year life cycle in the free-living state [21]. Additionally, most species of Ixodid ticks, including *H. longicornis*, are three-host ticks. The host's blood is used as the only source of energy during tick development. Only one blood-meal is taken during each life stage, and after completion of feeding ticks can survive for several months without a subsequent blood-meal. Because ticks have such unique feeding behavior, it is speculated that they are equipped with an efficient blood-digestion and nutrient-utilization system for survival [22]. However, details of the mechanisms by with ticks can survive for long periods without feeding are unknown. Knowledge of the mechanisms of feeding and reproduction are essential for the development of rational approaches to tick control.

Here, we provide the first report of tick LKR/SDH, its molecular cloning, characterization of the enzyme and determine the influence of lysine catabolized products on tick developmental stages.

## Results

### Structural features of LKR/SDH cDNA

The cDNA clone encoding *H. longicornis* LKR/SDH from the midgut cDNA library of *H. longicornis* was sequenced. The nucleotide sequence of LKR/SDH (GenBank accession no. AB464837) and deduced amino acid sequence are shown in supporting information (S) **Figure S1 and S2**. Sequence analysis shows that the LKR/SDH cDNA is 4,502 bp and consists of a 720 bp 5′-noncoding sequence, an open reading frame (ORF) of 2,811 bp and a 902 bp 3′-untranslated region. The 3′-untranslated region ends with a 19 bp poly (A) tail that begins 16 bp downstream from AATAAA, the eukaryotic consensus polyadenylation signal. The LKR/SDH cDNA has an ORF extending from position 721 to position 3,534 that codes for 937 amino acids with a predicted molecular mass of 104.0 kDa and theoretical isoelectric point (pI) of 6.63. No signal peptide was detected in the deduced amino acid sequence. Domain structure analysis reveals that the two lysine 2-oxoglutarate reductase domains and saccharopine dehydrogenase domain as detected with the SMART program (**Figure S1**). As shown in **Figure S2**, a search for potential promoter-related elements within the coding DNA region of the LKR/SDH gene revealed putative CAAT boxes at position 608 to 611 that encode the 5′-noncoding sequence region with a linker region between the LKR and SDH domains 2094 to 2097, a LKR domain from 2802 to 2805 and a SDH domain from 3342 to 3345. One endosperm box (E-box) was detected in the SDH domain region and 2 boxes detected in the 3′-untranslated region (**Figure S1**). No TATA box and Opaque 2 box were detected in the DNA sequence.

**Figure 1:**
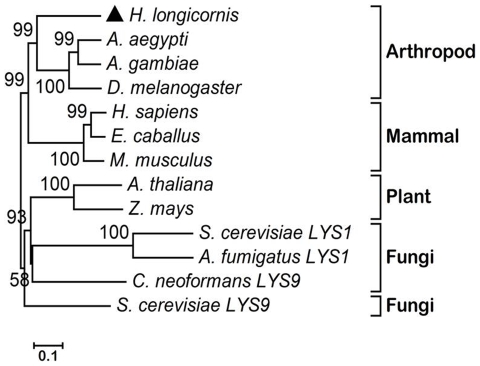
Phylogenetic tree of the protein sequences of LKR/SDH genes. The LKR/SDH amino acid sequences used were downloaded from GenBank and aligned in MEGA4 with CLUSTALW and the plylogenetic tree made by MEGA version 4.0 based on the neighbor-joining method. Values with 1,000 replications. The sequences used were *A. gambiae* (XP314728), *Aedes aegypti* (XP001649464), *D. melanogaster* (AAF52559), *H. sapiens* (CAA07619), *M. musculus* (CAA12114), *Equus caballus* (XP001502225), *Arabidopsis thaliana* (AAB53975), *Zea mays* (AAG21985), *Saccharomyces cerevisiae* LYS9 (CAA96331) (LYS9-L-glutamate forming), *Cryptococcus neoformans* (LYS9-L-glutamate forming) (AAW40810), *S. cerevisiae* LYS1 (CAA86194), (LYS1-L-lysine forming), and *Aspergillus fumigatus* LYS1P (XP754450) (LYS1-L-lysine forming).

A BLAST analysis revealed that *H. longicornis* LKR/SDH shares 62% identity with *Anopheles gambiae* (XP314728), 61% identity with *Drosophila melanogaster* (AAF52559), 53% identity with *Homo sapiens* (CAA07619), 53% identity with *Mus musculus* (CAA12114) and 52% identity with *Oncorhynchus mykiss* (AAU95502) (**Figure S2**). A phylogenetic tree using amino acid sequences of LKR/SDH from different sources by the neighbor-joining method verified the confidence of the branching order by 1,000 bootstrap replicates with the MEGA 4.0 software. The neighbour-joined trees revealed that *H. longicornis* LKR/SDH and mammalian bifunctional LKR/SDH represents a separate group from plants and monofunctional LKR and SDH of fungi. Interestingly, *H. longicornis* LKR/SDH is the most closely related to the mammalian-arthropod subgroup (**Figure 1**).

### Transcription analysis of the LKR/SDH mRNA by RT-PCR

**Figure 2:**
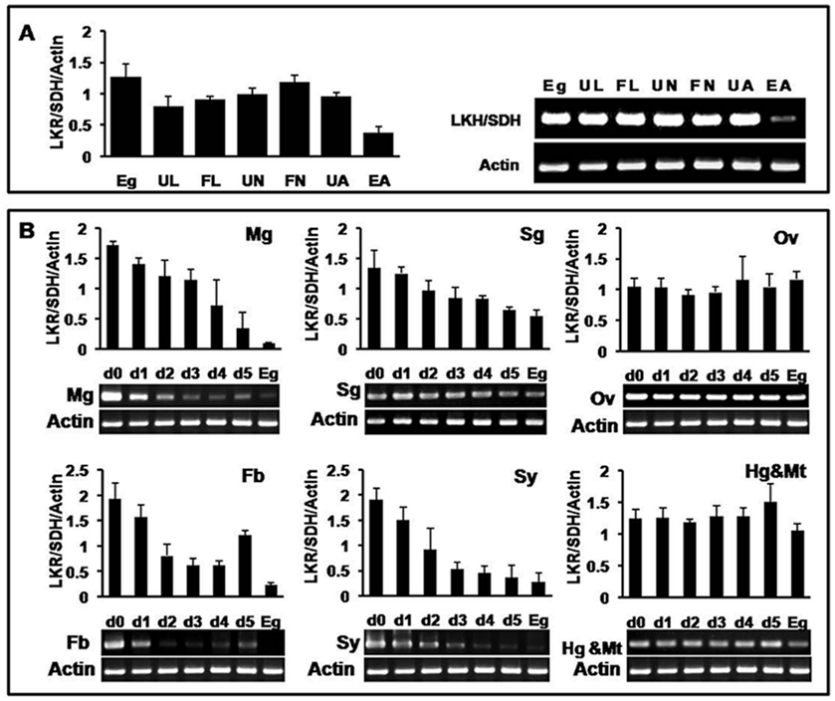
Transcription analysis of the LKR/SDH mRNA. The RT-PCR results shown are representative of 3 independent experiments with the same protocol. A, Stage-specific expression profiles of LKR/SDH shown by RT-PCR analyses. Lane 1, eggs; lane 2, unfed larvae; lane 3, fed larvae; lane 4, unfed nymphs; lane 5, fed nymphs; lane 6, unfed adult females, lane 7, engorged females of *H. longicornis*. RT-PCR was performed using LKR/SDH-specific primers and amplified 487-bp fragments. A 540 bp fragment of the constitutively expressed *H. longicornis β-*actin was amplified as an internal control. B, RT-PCR analysis of LKR/SDH mRNA expression in different tissues during blood feeding, Mg, midguts; Sg, salivary glands; Ov, ovaries; Fb, fat bodies; Sy, synganglions; Hg & Mt, hindguts with malphighian tubule. UF, unfed adult females; d1, females fed 1 day; d2, females fed 2 days; d3, females fed 3 days; d4, females fed 4 days, d5, females fed 5 days; Eg, engorged females.

To determine the expression profiles of the LKR/SDH gene, total RNA samples were extracted from different tick developmental stages. As shown in **Figure 2A**, similar levels of LKR/SDH mRNA transcripts were detected in all stages of ticks except for what appears to be mRNA down regulation by engorgement in fed females. All tissues including the midgut, salivary glands, ovaries, fat bodies, synganglion and hindguts with Malpighian tubules from both partially fed and fully engorged adult ticks showed expression of the LKR/SDH gene (**Figure 2B**). A gradual decrease in the expression of the LKR/SDH gene during feeding (day 1 to day 6) was observed in the midgut, salivary glands, fat body and synganglion. However, similar levels of expression of the gene were detected on all days investigated in the ovaries and hindgut with Malpighian tubules (**Figure 2B**). We found the transcript of the LKR/SDH gene was highly expressed in the midgut, ovary, fat body and synganglion of unfed ticks (**Figure 2B**).

### Expression of recombinant LKR, SDH, LKR/SDH in *E. coli* and Sf9 cells

**Figure 3:**
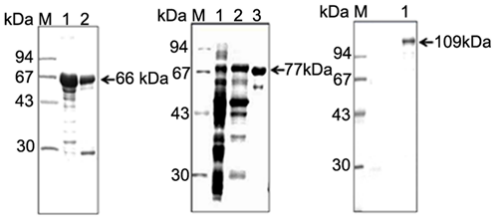
Expression of the recombinant protein. Recombinant proteins containing the LKR domain, SDH domain and full length LKR/SDH were resolved by 12% SDS-PAGE. A, lane 1, protein extracts from induced *E. coli* carrying pGEX-4T-3/LKR; lane 2, purified GST/LKR fusion protein; B, lane 1, lysate of *E. coli* without pGEX-4T-3; lane 2, protein extracts from induced *E. coli* carrying pGEX-4T-3/SDH; lane 3, purified GST/SDH fusion protein. C, lane 1, purified recombinant His-LKR/SDH protein; M, molecular weight marker.

The 1261 bp cDNA fragment encoding the LKR domain and 1311 bp cDNA fragment encoding the SDH domain of the LKR/SDH polypeptide were amplified by PCR. The LKR domain and SDH domain fragments were cloned into the pGEX-4T-3 vector. Problems occurred in insertion of the full ORF of tick LKR/SDH into a plasmid for GST fusion protein expression, so partial sequences encoding SDH and LKR domains were separately cloned and expressed in *E. coli* as two recombinant proteins, namely rGST/SDH and rGST/LKR. Successful expression of rGST/LKR and rGST/SDH fusion proteins in *E. coli* was confirmed by SDS-PAGE. The molecular masses of the rGST/SDH and rGST/LKR fusion proteins were estimated to be 74.0 kDa and 66.0 kDa containing a 26 kDa GST protein (Figures 3A and B). In the *E. coli* system, the expression levels of the recombinant proteins were high but the enzyme activity was low. Therefore, we used the BacMagic baculovirus expression system by placing a His tag at the N- terminus of the coding region of the genes to allow for rapid purification of over-expressed proteins by a Probond purification kit (Invitrogen) under denaturing conditions according to the manufacturer's instructions. The eluted recombinant full-length His-LKR/SDH exhibited a major single band with a molecular weight of 109 kDa on 10% SDS-PAGE (**Figure 3C**).

### Enzyme activity assay

**Figure 4:**
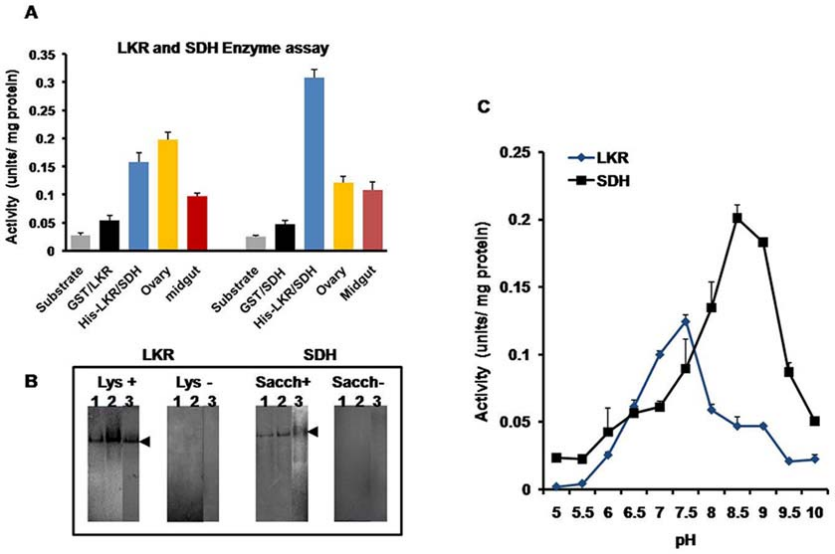
Enzymatic activity assay. A. Native and recombinant proteins were analyzed for LKR and SDH activities under conditions of excess concentrations of all LKR substrates or all SDH substrates, respectively. Data are presented as units/mg protein. Mean±S.D (n = 3). B. Separation of LKR and SDH activities by native-PAGE gel electrophoresis. Samples of partially purified midgut and ovaries, and recombinant His-LKR/SDH were separated by native PAGE and subsequently stained for SDH and LKR activity. In the control gel, samples incubated without L-lysine and saccharopine are labeled “Lys-” and “Sacch-”. In the LKR and SDH activity staining gel, the samples are labeled “Lys+” and “Sacch+”. Lane 1, midgut; lane 2, ovary; lane 3, His-LKR/SDH. C. Optimum pH of LKR/SDH native protein.

Enzymatic activities of LKR/SDH partially purified native proteins from midgut and ovarian lysates, and GST/LKR, GST/SDH and recombinant full-length His-LKR/SDH proteins were measured a spectrophometer (Figure 4A). LKR activity of partially purified native LKR/SDH protein from the midgut was 0.097 units/mg and SDH activity was 0.101 units/mg, whereas the LKR and SDH activities of native LKR/SDH protein from the ovaries were 0.194 and 0.121 units/mg, respectively. The enzymatic LKR activities of recombinant His-LKR/SDH fusion protein was 0.156 units/mg and GST/LKR fusion protein was 0.059 units/mg. Enzymatic SDH activity of His-LKR/SDH fusion protein was 0.304 units/mg and GST/SDH protein was 0.046 units/mg (Figure 4A).

The partially purified LKR/SDH enzyme from the midgut lysates, ovary lysates and His-LKR/SDH recombinant proteins were separated by non-denaturing gel electrophoresis and submitted to in-gel staining procedures to detect SDH and LKR activities. As shown in **Figure 4B**, the enzymatic activities analyzed by the native gel staining of native partially purified enzymes from the midgut and ovary extracts and recombinant His-LKR/SDH fusion protein showed both LKR and SDH activity. LKR/SDH enzyme partially purified from the tick midgut and ovary and recombinant His-LKR/SDH protein produced intensive black bands that were stained with the saccharopine and lysine substrate but no reaction in the absence substrates, indicating a specific activity for the LKR and SDH enzymes.

The specific activity of partially purified LKR/SDH native proteins of *H. longicornis* was optimal at pH 5.5–9.5 but was inactive under acidic (pH<5) or alkaline (pH>10) conditions. The optimum pH for LKR was 7.5 and for SDH was 8.5 (Figure 4C).

### Detection of native protein size

**Figure 5:**
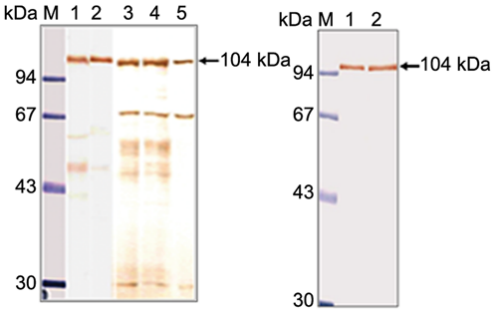
Immunoblotting analysis of native LKR/SDH. The partially purified LKR/SDH enzyme from midgut, ovary and egg lysates of partially fed adults, and whole body lysates of larvae, nymphs, unfed adult, and fed adult *H. longicorns* were used as antigens. A. Lane M, protein marker; lane 1, egg lysate; lane 2, larva lysate; lane 3, nymph lysate; lane 4, unfed tick lysate; lane 5, engorged tick lysate. B. Lane M, protein marker; lane 1, adult midgut; lane 2, adult ovary.

To determine the molecular weight of endogenous native LKR/SDH protein corresponding to the cloned cDNA product, mouse anti-rGST/SDH serum was used to probe the immunoblot. The partially purified LKR/SDH proteins from the midgut, ovary and eggs from partially fed adults and whole body lysates from fed larvae, fed nymphs, and unfed and fed adult ticks were used for immunnoblotting. As shown in **Figure 5**, a specific strong band of approximately 104 kDa was detected in the eggs from partially fed adults, and larval, nymphal, unfed female and engorged female lysates (**Figure 5A**) and also in the midgut and ovaries from partially fed adults (**Figure 5B**). No band was detected for the control anti-rGST serum in any of the samples (data not shown). These results show that all developmental stages as well as the midgut and ovaries of partially fed adult ticks express the LKR/SDH protein. Two additional faint bands were also detected in the samples of **Figure 5A**, probably due to degradation of the full-length LKR/SDH protein.

### Indirect fluorescent antibody test (IFAT)

**Figure 6:**
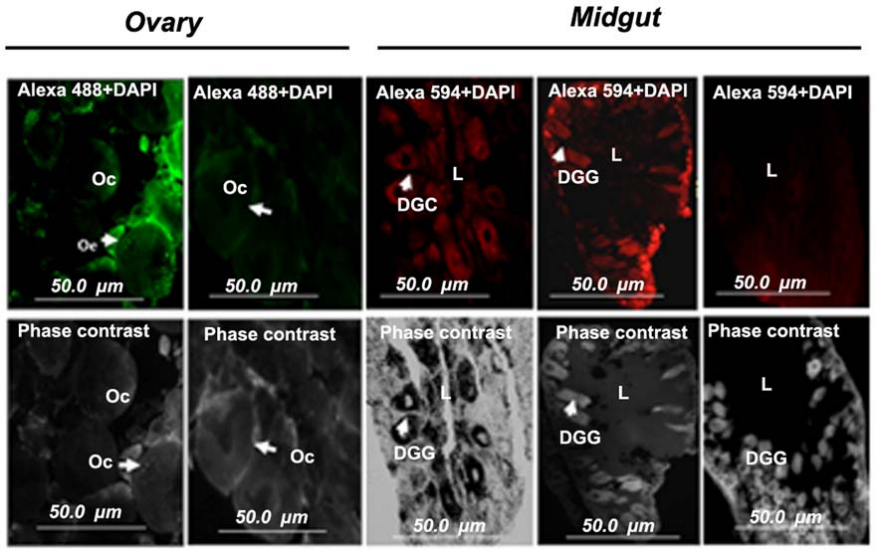
Localization of LKR/SDH in ovary and midgut cells by IFAT. Slides of ovary or midgut tissue from adults 4 days post-engorgement were used with mouse anti-SDH serum as primary antibodies. The mouse anti-IgG conjugated with Alexa 488 was used as a secondary antibody for ovarian tissues and mouse anti-IgG conjugated with Alexa 594 for midgut tissues. DGC, digestive cell; Oc, oocyte; L, midgut lumen. Magnification, ×20.

To examine LKR/SDH localization in the ovary and midgut of 4 day post-engorgement adult ticks, an indirect fluorescent antibody test was performed using anti-GST/SDH immune serum as test serum and anti-GST mouse serum as negative control. The Alexa 488- and Alexa 594-conjugated anti-mouse immunoglobulins were used as second antibodies. As shown in **Figure 6**, strong positive fluorescence was observed in the ovary oocytes and midgut digestive cells, indicating the endogenous enzymes were expressed in these cells.

### Histochemical detection of SDH activity in sectioned midgut and ovary

**Figure 7:**
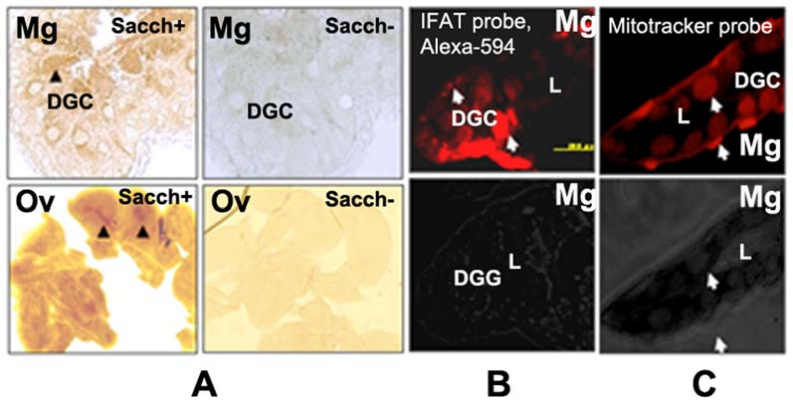
Histochemical detection of SDH activity in sectioned midgut and ovary. A. In the SDH activity staining tissue, slides stained with saccharopine are labeled with “Sacch+” and control slides not incubated with saccharopine labeled with “Sacch-”. Positive reaction shows SDH specific staining as brown dots on the oocytes and digestive cells of the midgut, whereas negative reaction incubated without saccharopine shows no specific staining. Mg, midgut; DGC, digestive cell; L, lumen; Ov, Ovary; Oc, oocyte. B. IFAT; The mouse anti-IgG conjugated with Alexa 594 was used as a secondary antibody. DGC, digestive cell; L, lumen; C. Mitochondria visualization by MitoTracker probe. MitoTracker probe, midgut tissues stained with 50 nM of Mitotracker Red CMXRos. DGC, digestive cell; L, midgut lumen. Magnification, ×20.

We analyzed the ovary and midgut of adult ticks by *in situ* histochemical staining for SDH activity. As shown in **Figure 7**, SDH specific enzymatic activity was detected in the oocytes and the digestive cells of the midgut. The staining showed a specific reaction with experimental sections but not with control sections incubated in the absence of saccharopine, indicating that the reaction is specific for SDH and not due to spurious detection of NAD^+^ by dehydrogenases. As indicated in **Figure 7A**, specific reactions were observed as small spots near the nuclei and in the central part of the oocytes. Moreover, these strong reactions were concentrated in the regions of the midgut digestive cells. Mitochondria, visualized by the highly-specific probe MitoTracker, exhibited a pattern almost identical to the LKR/SDH labeling by IFAT (**Figure 7B and C**). Our immuno-histochemical and MitoTracker probe results showed that LKR/SDH enzyme of this tick is localized in the cytosol and mitochondria (**Figure 6** **and** **7**).

### Effect of starving conditions and lysine or saccharopine injections

**Figure 8:**
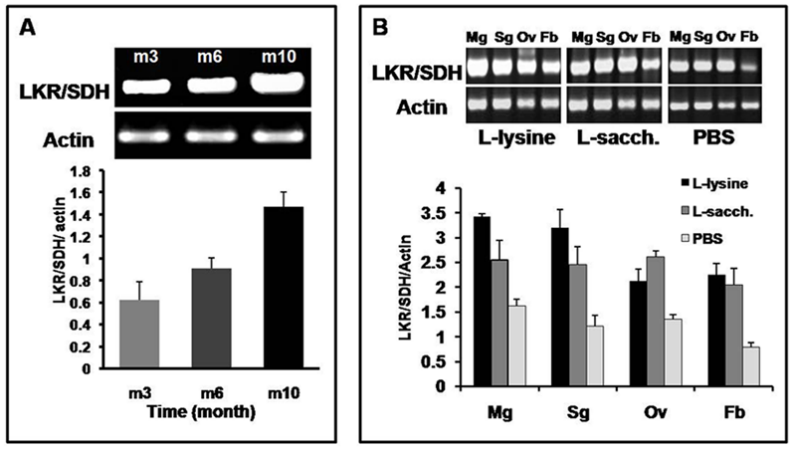
Expression profiles in tick starvation and after injections of lysine and saccharopine. Total RNA was pooled from the same concentration of RNA samples from starved ticks. The RT-PCR results shown are representative of 3 independent experiments with the same protocol. A. m3, females starved for 3 months; m6, females starved for 6 months; m10, females starved for 10 months. B. Effect of injections of synthetic L-lysine and saccharopine on LKR/SDH gene expression. Ticks starved for 1 month were injected with synthetic L-lysine (50 mM solution of L-lysine 0.5 µl/tick), L- Saccharopine (20 mM solution L-saccharopine 0.5 µl/tick) and PBS (0.5 µl/tick), respectively. Mg; midguts; Sg, salivary glands; Ov, ovaries; Fb, fat bodies. The data are expressed as the ratio of the density of LKR/SDH to the density of actin gene products from the same template. The RT-PCR results shown are representative of 3 to 4 independent experiments with the same protocol.

Consistent expression of LKR/SDH was detected in all developmental stages, indicating an important role throughout the life cycle of a tick, including the long period of starvation after detachment from the host. The expression was up-regulated in unfed ticks (**Figure 8A**) and up-regulation corresponded to the length of starvation. This result indicates that the LKR/SDH gene plays a crucial role in regulation of physiological conditions during starvation in ticks. To investigate the role of this gene and impact of lysine level in tick physiology, we injected lysine or saccharopine into the tick haemocoel. After 18 hours of incubation at 25°C, these ticks were allowed to feed on rabbits. The ticks were removed after 3 days of blood-feeding and total RNA extracted. RT-PCR showed that the LKR/SDH mRNA level was up-regulated in ticks injected with lysine or saccharopine but not the controls injected with PBS (**Figure 8B**).

### Gene silencing of LKR/SDH by RNA interference

**Figure 9:**
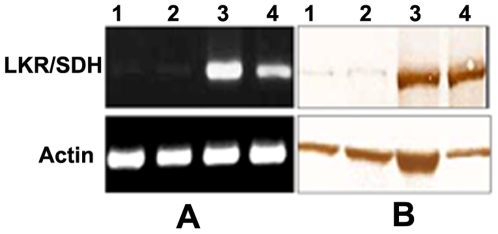
Effect of dsRNA treatment on LKR and SDH gene disruption. A. Reverse transcription PCR analysis. B. Western blotting analysis. LKR-, SDH- and Luc- dsRNAs and PBS were injected into the each group of female *H. longicornis* adults. The injected ticks (15 individuals for each group) were infested on rabbits, and ticks recovered after 4 days of feeding. Lane 1, LKR dsRNA-injected ticks; lane 2, SDH dsRNA-injected ticks; lane 3, PBS-injected ticks; lane 4, firefly gene of Luciferase (Luc) dsRNA-injected ticks.

The reduction of LKR/SDH expression by RNAi was demonstrated at the mRNA and protein levels by RT-PCR and Western blot, respectively (**Figure 9**). Gene specific primers were used for RT-PCR transcriptional analysis, and parallel RNA samples with actin-specific primers were amplified as positive controls. The LKR dsRNA and SDH dsRNA-treated groups showed a considerably greater decrease in LKR/SDH transcription than the control group (**Figure 9A**). Western blot analysis showed that LKR/SDH protein expression was clearly reduced in LKR and SDH dsRNA-injected ticks when compared to the PBS and Luc dsRNA-injected control ticks (**Figure 9B**).

### Phenotypic effects of LKR/SDH gene silencing on feeding, oviposition and osmoregulation

The phenotypic differences between the dsRNA-injected and control groups was investigated by measuring the attachment rate, the blood feeding periods, total number of ticks engorged, engorged body weight, mortality, pre-oviposition period, feces excretion, oviposition, weight of egg mass per tick and percent of eggs hatching. No significant differences were observed between the experimental and control groups in attachment rate and engorgement percentage. However, reproductive phenotype, shown as blood feeding periods, engorged body weight, pre-oviposition period, oviposition period, weight of egg mass per tick and egg-hatching rate were significantly affected by LKR- and SDH- dsRNA treatments (**Table 1**).

**Table 1:** Effect of dsRNA treatment on tick oviposition.

Group	*N*	Blood feeding period (day)	Engorged body weight (mg)	Pre-oviposition period (day)	Weight of egg mass (mg/tick)	Egg viability (%)
PBS	45	4.0–5.0	316.5±33.5	5.0–6.0	166.0±14.6	100%
dsRNA/Luc	50	4.5–6.5	279.4±57.3	5.0–6.0	147.2±34.0	100%
dsRNA/SDH	50	6.5–7.5	246.8±64.8	6.5–10.0	109.8±36.5*	49%*
dsRNA/LKR	50	6.5–7.0	201.0±54.7*	NO	0.0	0.0%

N, Number of ticks, PBS, PBS-injected ticks; dsRNA/Luc, dsRNA/Luc-injected ticks, dsRNA/SDH, dsRNA/SDH-injected ticks; dsRNA/LKR, dsRNA/LKR-injected ticks; NO, not-observed.

* P<0.001, significantly different calculated by Student's *t* test.

The blood feeding periods of the SDH dsRNA-injected group (6.5–7.5 days) and LKR dsRNA-injected group (6.5–7.0 days) were 2–3 days longer than the Luc dsRNA- (4.5–6.5 days) and PBS- (4–5 days) injected control groups. Body weight of engorged ticks that were treated with dsRNA of the SDH domain significantly decreased (p<0.001) compared to the control groups. Egg weight also significantly decreased (p<0.001) in the treated group compared to the two control groups. As shown in **Table 1**, pre-oviposition period of SDH-dsRNA-injected ticks was 6.5–10 days, which was 1.5–4 days longer than the control groups. Therefore, significant reduction in reproduction occurred in SDH dsRNA-injected ticks (**Table 1**). Percentage of eggs that hatched was 49% (p<0.001) of all eggs laid by the SDH dsRNA-injected ticks compared to 100% for the control groups. SDH gene silencing appears to greatly effect oviposition by female ticks.

LKR dsRNA-injected ticks showed significantly reduced (28–36%) engorged body weight compared to the control groups. Ovipositioning was not observed in any ticks treated with LKR dsRNA (**Table 1**). A 2–4 times greater volume of hemolymph (25.3±4.0 µl) was collected from LKR dsRNA-injected ticks than the control groups (data not shown).

**Figure 10:**
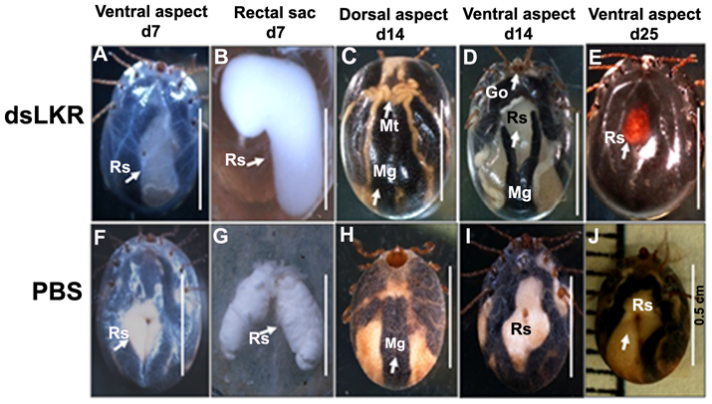
Morphological changes in LKR silenced ticks. The macrophotographs of dsLKR RNA-injected tick (above) and PBS-injected tick (below). A, Ventral aspect of dsLKR RNA-injected ticks 7 days after engorgement, Rs, rectal sac (arrow). B, Magnification of A and rectal sac of dsLKR RNA-injected ticks show significant enlargement due to filling with fluid; C and *D*, Dorsal and ventral aspects of dsLKR RNA-injected tick 14 days after engorgement,show edema-like condition and hernia-like protrusion of Gene's organ. Mg, midgut; Mt, Malpighian tubule, Rs, rectal sac; Go, Gene's organ; E, Ventral aspect of dsLKR RNA-injected tick 25 days after engorgement, Rs, rectal sac changed color from white to red (arrow); F to J; PBS-injected tick. F, Ventral aspect of PBS-injected tick 7 day after engorgement; G, Magnification of F and rectal sac; H, Dorsal aspect 14 days after engorgement; I, Ventral aspect 14 days after engorgement; J, tick after egg oviposition 25 days after engorgement.

**Figure 11:**
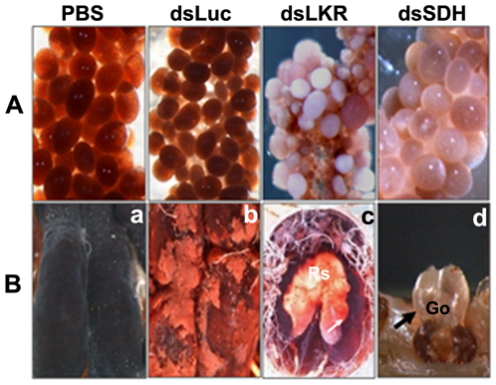
Microphotographs of dsRNA-injected tick and macro photograph of pathological organs. A. Ovaries of PBS (normal), LKR and SDH dsRNA-injected ticks showing pathological phenotypes. B. Macrophotograph of pathological organs. Panel a, Normal midgut of PBS-injected ticks on day 25 after engorgement, Mg, midgut; panel b, Destroyed structure and appearance of liquid in the midgut of LKR dsRNA-injected tick on day 25 after engorgement, Mg, midgut; panel c, Magnification of Figure 11E, rectal sac of LKR dsRNA-injected tick on day 25 after engorgement shows significant enlargement due to filling with fluid-like redden liquid, Rs, rectal sac; panel d, Hernia-like protrusion of Gene's organ, LKR dsRNA-injected tick on day 14 after engorgement, Go; Gene's organ.

Interestingly, morphological and functional changes occurred in the rectal sac, midgut, hindgut, ovary, Gene's organ and Malpighian tubules of LKR dsRNA-injected engorged ticks (**Figures 10** **and** **11**). Pathomorphological examination showed that the midgut, Malpighian tubules and rectal sac were filled with a watery liquid and the tick cuticle was stretched and thinned due to a large amount of water in the female ticks. This high volume of hemolymph and hydraulic pressure made the tick cuticle transparent so all the internal organs were easily visible through the cuticle. In addition, the Gene's organ protruded with a hernia-like morphology probably due to the high hydraulic pressure from the large volume of hemolymph (**Figures 11B, d**). Lower amounts of guanine crystals were observed in the rectal sac and Malpighian tubules. None of these dramatic changes were observed in ticks from the 2 control groups (**Figures 10** **and** **11**).

Oocytes developed normally in PBS-injected and Luc dsRNA-injected ticks, but oocytes of LKR dsRNA- and SDH dsRNA-injected ticks were white or light brown and smaller in size, indicating immature stages of development even 7 days after engorgement (**Figures 11A**). Furthermore, the LKR dsRNA-injected engorged ticks did not oviposit and started to die within 25 days after engorgement. The dead ticks were dissected and morphological changes observed. The midgut and hindgut increased in size, structures were modified and liquid appeared in the gut likely because of osmotic or water balance stress. Moreover, the midgut, Malphigian tubules and rectal sac of dissected ticks were filled with a red liquid and immature oocytes were observed. However, these changes were not observed in the 2 control groups. The effects of LKR/SDH gene silencing on feeding, oviposition and osmoregulation indicate lysine catabolism plays a critical role in the regulation of these processes in ticks as well as plants and mammals.

## Discussion

Lysine catabolism has received considerable attention, due to novel findings on the characterization and regulatory aspects of the bifunctional enzyme LKR/SDH. The LKR/SDH has been reported from diverse sources, including plants and mammals [6], [7], [10], [23], but to our knowledge its activity and biological functions are little known in any arthropods [24]. Therefore, in the current study, we have isolated and characterized a gene encoding LKR/SDH from a midgut cDNA library of partially fed *H. longicornis* ticks as the first approach towards understanding the physiological role of the lysine degradation pathway in ticks. This gene encodes a bifunctional polypeptide bearing domains similar to the plant and mammalian LKR/SDH enzymes [7], [10], [25].

The bifunctional protein encoded by the LKR/SDH *H. longicornis* gene possesses 937 amino acid residues and is divided into three distinct regions: an N terminal domain similar to LKR; a C-terminal domain similar to SDH; and an interposed short region connecting both domains that show similarities to other known protein sequences in the databases (**Figure S1 and S2**). The interposed region contains promoter related CAAT box and an identical short region similar to mammals but distinctive from plants and other animals. This region is short in arthropods and mammalians (**Figure S1**) but longer in maize and *Arabidopsis* (comparison not shown) [10]. This result is similar to that previously reported by Papes et al. [10], in which the LKR and SDH domains of the predicted protein present strong similarity not only to the mammalian enzymes (**Figure S1 and S2**) but also to the LKR and SDH domains of bifunctional proteins from maize and *Arabidopsis*. A phylogenetic analysis revealed that *H. longicornis* LKR/SDH clustered into a branch nearest the mammalian LKR/SDH members (**Figure 1**). This indicates that LKR/SDH of *H. longicornis* possesses a similar function to that of plants and mammals. The predicted molecular mass of this protein is 104 kDa, which is similar to the 109 kDa protein reported for the enzyme purified from mouse and 115 kDa reported for the enzyme purified from bovine liver [10], [26]. The protein isolated from mammals is a tetramer whereas in plants it is a dimer [25], [27]. The tetramer and dimer structures were not observed in the bifunctional LKR/SDH of *H. longicornis*.

The LKR/SDH gene was expressed in all developmental stages of the hard tick and also in all tested internal organs. However, the mRNA levels were down-regulated in engorged adult ticks and the expression of the LKR/SDH gene gradually decreased in the midgut, salivary gland, fat body and synganglion during blood feeding in adult ticks. However, the LKR/SDH mRNA was consistently expressed at the same levels in the ovary and hindgut with Malpighian tubules (**Figure 2B**), and protein expression was higher in the ovary than other organs (**Figure 5B**). The high expression level observed in the ovarian tissues is consistent with activity observed in ovarian tissues of plants and humans [2], [7], [14], [28]. The LKR-SDH gene is abundantly expressed in floral tissues and seeds of plants during development. LKR/SDH appears to play an important role in egg production and reproduction. Accordingly, this enzyme may also be involved in these pivotal physiological processes and embryonic development of ticks.

The partially purified native protein and recombinant GST/LKR fusion protein, recombinant GST/SDH fusion protein and recombinant His-LKR/SDH full-length fusion protein showed both LKR and SDH activities by native gel staining and spectrophotometry. The native enzyme from ovaries and His-LKR/SDH full-length fusion protein expressed in the baculovirus expression system showed higher enzymatic activities than the recombinant GST/LKR domain and GST/SDH domain fusion proteins. Another interesting property of LKR/SDH is that the two linked enzymes possess significantly different pH activity optima with maximal activity of LKR at a neutral pH and of SDH at pH 9 or higher [25], [29]. The optimum pH for enzymatic activity is similar to previous reports for plants and mammals [8], [26].

The LKR/SDH enzyme appears to be expressed in different cell compartments. The mammalian LKR and SDH enzymes are located in the mitochondrial fraction [27], [30], [31] whereas plant LKR/SDH is located in the cytosol [14], [32]. The immuno-histochemical and MitoTracker probe showed that the tick LKR/SDH enzyme is localized in the cytosol and mitochondria in midgut and ovary similarly to plants and mammalian LKR/SDH. We found the transcript of the LKR/SDH gene was highly expressed in the midgut, ovary, fat body and synganglion of unfed ticks (**Figure 2B**). In addition, expression of LKR/SDH was detected in all developmental stages indicating an important role throughout the life cycle of the tick, including a long period of starvation after detachment from the host. The expression of LKR/SDH was up-regulated in unfed ticks and this up-regulation corresponded to the length of starvation (**Figure 8A**). Moreover, Papes et al. [10], observed an 82% increase in LKR mRNA and 52% increase in LKR activity in mice starved 1–2 days. Lysine is a ketogenic amino acid so degradation can be coupled to the energetic processes during limited carbon supplies such as starvation. In the liver, LKR/SDH is thought to participate in regulating the nitrogen and energetic balances by controlling the conversion of lysine into α-aminoadipic semialdehyde, which is then converted into ketone bodies by several reactions. These energetic compounds can then be used in situations of limited carbon supply [10]. Glutamate and α-aminoadipic semialdehyde are dominant products of the lysine catabolism pathway and serve as major precursors for synthesis of glutamine, proline and L-pipecolic acid [33]–[35]. The fat body in arthropods is an organ that stores food reserves, metabolizes hormones and other essential messenger molecules and detoxifies wastes or harmful compounds. Its functional value to the animal has been compared to that of the vertebrate liver [35]. In these organs, LKR/SDH is thought to participate in the nitrogen and energetic balances by controlling the degradation of lysine in the tick. L-pipecolic acid inhibits food intake and induces sleep-like behaviors in the neonatal chick. L-PA is activated by both GABA-A and GABA-B receptors, GABA-B receptors alone contribute to food intake whereas both receptors contribute to sleep-like behavior [36], [37]. Galun and Kindler [38], observesd that glutamic acid also inhibited feeding in a tick. These results indicate that LKR/SDH gene and lysine degradation pathways play a crucial role in the regulation of the physiological condition of ticks during starvation.

The Lys catabolism pathway is an important route for balancing Lys levels, mostly because high Lys is toxic to plants and mammalian cells [8], [27], [39]. In particular, high-lysine or high-protein diets promote increases in LKR and SDH activities in the rat liver [11], [40]. Also, LKR and SDH activities as well as LKR/SDH mRNA increase in mice receiving lysine or saccharopine injections or diets containing excess lysine [12], [39]. To investigate the role of this gene in tick physiology and the impacts of lysine levels, we injected lysine or saccharopine into ticks. LKR/SDH mRNA levels increased in the midgut, salivary gland and fat body of lysine or saccharopine injected ticks (**Figure 8B**). These results indicate LKR/SDH enzymes control free lysine levels in these organs of the tick. In mammals, it seems relevant because physiological or pathological lysine accumulating or limiting conditions may seriously affect the lysine degradation pathway, leading to restricted or enhanced catabolism. According to Papes et al. [10], lysine catabolic pathway can respond promptly to and influence both the nitrogen and carbon balances in the organism. Therefore, we conclude that LKR/SDH gene regulates the lysine levels in ticks.

The functional role of LKR/SDH is unclear in ticks. RNA interference (RNAi) is becoming a powerful post-transcriptional gene silencing technique that provides insight into gene function [41]. To investigate the function of LKR/SDH in ticks we constructed small dsRNAs containing LKR or SDH domains and used them for RNA silencing experiments. The RNA interference of LKR or SDH domains showed degradation of amino acids and a clear influence on egg production and tick reproduction. Morphological and functional changes occurred in the rectal sac, midgut, hindgut, ovary, Gene's organ and malphigian tubules of LKR dsRNA-engorged ticks. In addition, the Gene's organ protruded with a hernia-like morphology, and the glandular portion did not revert to the normal position (**Figure 11B, d**). These changes may be due to the silenced LKR domain causing high hydraulic pressure in the hemolymph. During oviposition in ticks, each egg is coated with a waterproofing wax secreted by the Gene's organ. Gene's organ consists of a glandular portion and a set of rectractor muscles. Booth et al. [42], suggested that reversion of the glandular portion is a function of the haemolymph hydrostatic pressure and effected by the retractor muscles. The application of L-glutamate to the retractor muscles of the Gene's organ of *Amblyomma varlegatum* caused dose-dependent depolarizing responses [42], suggesting these are innervated by glutaminergic neurons [21]. Lysine is an important precursor for novo synthesis of glutamate, the most significant excitatory neurotransmitter in the mammalian central nervous system. The widespread distribution of the lysine degradation enzyme LKR/SDH in the mammalian central nervous system indicates that lysine is an important precursor of the neurotransmitter glutamate (via LKR/SDH) in several brain regions of adults as well as embryos [33], [43]. Glutamate may have neurotransmitter or neuromodulatory effects in ticks also.

Moreover, body size gradually increased after blood feeding due to imbalance of the osmotic regulation in LKR dsRNA-injected ticks and all died 25 days after engorgement due to irreversible pathological changes (**Figures 10** **and** **11**). In arthropods the Malpighian tubules, midgut and hindgut are the major organs for salt and water balance [44]. The Malpighian tubules and rectum of insects form a physiological complex that serves as a functional kidney at the organismal level. Primary urine is formed in the Malpighian tubules, followed by movement to the rectum where selective reabsorption of ions and water takes place. These secretion and reabsorption processes are responsible for maintaining osmotic balance between the intracellular and extracellular compartments [45]. The role of LKR/SDH in the kidneys is to influence the lysine reabsorption in the tubule cells [10]. The expression patterns shown in the RT-PCR analysis and also the pathological changes and dysfunction in the midgut, Malpagain tubule and rectal sac observed the RNA interference experiments may occur because of an osmotic imbalance. Morphological and functional changes occurring in the rectal sac, midgut, hindgut, ovary, Gene's organ and Malphigian tubules in engorged ticks of LKR dsRNA-injected ticks, (**Figures 10** **and** **11**) are evidence to support our hypothesis. This hypothesis provides important information about the similar functions of LKR/SDH enzymes regulating osmotic stress reported in plants only so far. [3], [4], [13], [14]. In plants, the LKR level was shown to be significantly up-regulated in inflorescence tissues and developing seeds, as well as in response to osmotic stress [3], [4], [15], [16]. Other studies report, a significant differential increase in LKR activity when the osmotic stress becomes more severe [15], [16]. In rapeseed, the LKR/SDH gene is the most responsive to osmotic stress [13], [16], [46]. Glutamate plays an important role in the body's disposal of excess or waste nitrogen and is the most abundant swift excitatory neurotransmitter in the mammalian nervous system. In crustaceans, FAA functions are associated with the processes of molting, growth, metamorphosis, osmotic regulation (i.e., osmoregulation) [47], and intracellular osmotic regulation (maintenance of the osmotic equilibrium between cells and the hemolymph) [47], [48]. LKR/SDH enzyme may serve to regulate lysine homeostasis in the tick tissues while efficiently converting lysine to glutamate and then to other osmotic-related metabolites in response to osmotic stress and certain developmental processes.

In conclusion, the results of this study show the LKR/SDH gene plays an important role in egg production, reproduction and development of the tick and also indicates an interesting role in carbon, nitrogen and water balance that is crucially important for development and survival of the tick. Finally, LKR/SDH enzymes play important and pivotal roles in tick physiology similar to other animals and plants.

## Materials and Methods

### Tick and animals

The parthenogenetic Okayama strain of *H. longicornis* has been maintained by feeding on ears Japanese white rabbits (Kyudo) for several generations in our laboratory since 1997 [18], [19]. Rabbits were cared for in accordance with the guidelines approved by the Animal Care and Use committee (approval no. A08010) of Kagoshima University.

### Identification and characterization of the cDNA encoding LKR/SDH

LKR/SDH was identified from expressed sequence tags (EST) constructed from midgut cDNA libraries of *H. longicornis*. The plasmids containing LKR/SDH gene-encoding inserts were extracted using the Qiagen DNA Purification kit (Qiagen). The nucleotide sequences of the cDNAs were determined by the big dye terminator method on an ABI PRISM 3100 automated sequencer (Applied Biosystems). Genetyx DNA analysis software (Genetyx) was used to deduce the amino acid sequence of LKR/SDH and the BLAST Program [49], for alignment was used to compare this sequence with previously reported sequences available in GenBank [50]. Theoretical molecular weights and isoelectric point (pI) were determined by using PeptideMass (http://us.expasy.org/tools/peptide-mass.html) [51]. Phylogenetic trees were generated from homologies of the LKR/SDH amino acid sequences from different sources by the neighbor-joining method, and the confidence of the branching order was verified by making 1,000 bootstrap replicates using the fourth version of MEGA software. The tree was viewed and converted to graphic format with TREEVIEW (http://taxonomy.zoology.gla.ac.uk/rod/treeview.html).

### RNA isolation and Semiquantitative RT-PCR expression analysis

Total RNA was extracted from whole bodies of ticks at each developmental stage and from dissected salivary glands, midgut, ovary, fat body with trachea, and hemolymph of unfed, partially engorged and fully engorged ticks using TRI reagent (Sigma-Aldrich) to determine stage and tissue distribution profiles of LKR/SDH. Single-strand cDNA was generated by reverse transcription using the transcriptor first strand cDNA synthesis kit (Roche Biochemicals) as recommended by the manufacturer. PCR was carried out with the appropriate dilutions of templates using LKR/SDH-specific primers (sense primer, 5′-CTGGGTCAACAGGACAACCT-3′; anti-sense primer, 5′-ACGCGGCCGCTCTACAA CGAATTCCCTC-3′). Control amplification was carried out using the specific primers (sense primer, 5′-CCAACAGGGAGAAGATGACG-3′; anti-sense primer, 5′-ACAGGTCCTTACGGATGTCC-3′) designed from *H. longicornis β*-actin (accession no. AY254898). A series of RT-PCR were performed in 50 µl of a mixture containing 1 µg of RNA, 100 pmol of oligonucleotides, 1x one-step RNA PCR buffer, 5 mM of MgCl_2_, 1 mM of dNTPs, 1 U of RNase inhibitor, 5 U of AMV RTase XL and 5 U of AMV-optimized Taq DNA polymerase. The reverse transcription reaction was carried out at 50°C for 30 min, and then PCR repeated for 35 cycles under the following conditions: 30 s of denaturation at 94°C, 1 min of primer annealing at 65°C, and 2 min of elongation at 72°C, all subsequent amplifications were therefore carried out using this cycle range and conditions. The PCR products were subjected to electrophoresis in a 1.5% agarose gel in TAE buffer; the DNA was visualized by ethidium bromide staining and analyzed using Quantity One 1-D Analysis Software (Quantity One Version 4.5, Bio-Rad Laboratories), in which band intensity is expressed in pixels. The housekeeping gene of *H. longicornis β*-actin was used as an internal expression control. Our preliminary data showed that *β*-actin did not change according to the stage of the tick or across tissues. Relative gene expression was calculated as the ratio of the band intensity of the cloned gene to that of *β*-actin.

### Construction of recombinant transfer vector for baculovirus expressing H. longicornis LKR/SDH cDNA

The 2811 bp PCR fragment from *H. longicornis* LKR/SDH containing the open reading frame was inserted into the *Not*I site of an AcNPV transfer vector pIExBac-3 (Novagen). Restriction enzyme analysis was performed to identify the construct containing the insert in the correct orientation. The construct was cut first with *Not*I to confirm the correct insert size. Cutting with *Bam*HI and *Kpn*I confirmed that the construct orientation was correct.

### Cell culture, DNA transfection and virus stock production

*Spodographa frugiperda* (Sf9) cells were grown in Grace's medium supplemented with 10% fetal bovine serum (Invitrogen) at 27°C. Sf9 cells were seeded in 35 mm tissue culture plates at 0.9×10^6^ cells in 2 ml of growth media. The cells were allowed to attach for 1 h, the medium was removed and the cells were washed twice with Grace's unsupplemented medium. Sf9 cells were co-transfected with recombinant transfer vector pIExBac-3-LKR/SDH and linear *Bac*Magic DNA (Novagen) with the Insect GeneJuice Transfection reagent (Novagen) and added to 1 ml unsupplemented medium. After 5 h the unsupplemented medium was removed and 2 ml of Grace's medium supplemented with 10% fetal bovine serum added to the plates. The SF9 cells were incubated at 27°C for 5 days. The culture medium containing recombinant virus was harvested, filtered and stored at 4°C. The expression of LKR/SDH in the Sf9 cells was confirmed by IFAT and Western blots with anti-SDH serum produced in mice immunized with recombinant GST/SDH expressed in *Escherichia coli*. After 2 cycles of amplification, a recombinant LKR/SDH virus stock was prepared and used to infect monolayer's of Sf9 cells for expressing the proteins in large-scale.

### Purification of recombinant LKR/SDH protein by infection of Sf9 cells using recombinant baculovirus

Sf9 insect cells were infected with the recombinant LKR/SDH virus. Cells were harvested 4 days post-infection and washed twice in ice-cold PBS. All the subsequent purification steps were performed at 4°C. The recombinant protein was a fusion protein with a poly-histidine tag and was purified using a ProBond purification kit (Invitrogen) under denaturing conditions according to the manufacturer's instructions. The purity of the protein was determined by SDS-PAGE with Coomassie Blue staining and Western blot analyses using the anti-truncated SDH domain of LKR/SDH mouse sera. The protein concentration was determined by the Micro BCA^TM^ protein assay kit (Pierce).

### Expression of truncated recombinant LKR domain and SDH domain proteins in *E. coli*

Fragments corresponding to the LKR and SDH domains were amplified by PCR with the primers 5′-ACGCGGCCGCGTGTTGGTGCTTGGAGCG-3′ and 5′-ACGCGGCCGCTCTACAACGAATTCCCTC-3′ for SDH fragment and the primers 5′-ACGAATTCA GCCGTGAGGGACGCC-3′ and 5′-ACGAATTCCTGTTGGCATGTTGTC-3′ for LKR together in PCR buffer, dNTP and Taq polymerase. The purified PCR product of the SDH domain was inserted into the *Not*I site of the plasmid pGEX-4T-3 and the purified PCR product of the LKR domain was inserted into the *Eco*RI site of the plasmid pGEX-4T-3 and then expressed as a glutathione-*S*-transferase (GST) fusion *protein* in *E. coli* (DH5α strain) according to the manufacturer's instructions (Amersham Pharmacia Biotech). The recombinant rGST/LKR and rGST/SDH fusion proteins were purified using Glutathione Sepharose 4B (GE Healthcare) according to the manufacturer's instructions. Determinations of the recombinant fusion protein concentrations were performed by SDS-PAGE and compared to known concentrations of the standard BSA protein (Pierce).

### Production of anti-LKR/SDH Sera

Female mice (ddY, 7 weeks old) were immunized intraperitoneally three times at 2 week intervals with 100 µg of recombinant GST/SDH domain fusion protein in Freund's adjuvant (Sigma). Sera were collected from these mice 10 days after the last immunization. Mice were cared for in accordance with the guidelines approved by the Animal Care and Use committee (approval no. A-08010) of Kagoshima University.

### Partial purification of LKR/SDH native proteins from tick midgut and ovaries

*H. longicornis* midgut and ovarian tissues were used for preparation of partially purified native protein using the method described by Papes et al. [10], with some modifications. The following steps were then performed at 4°C, one part of tissue was homogenized in 5 parts of homogenization buffer A (25 mM sodium phosphate (pH 7.4), 1 mM EDTA, 5 mM DTT) containing 5 mM benzamidine and 100 µM leupeptin. The homogenate was centrifuged for 5 min at 400 *g* and the pellet discarded. The supernatant was centrifuged at 20,000 *g* for 15 min. The pellet was resuspended in homogenization buffer and recentrifuged at 20,000 *g* for 15 min. The washed pellet was suspended in 3–4 volumes of homogenization buffer and again disrupted with a glass homogenizer. The pH of the supernatant was adjusted to 5.6 with the addition of solid NaH_2_PO_4_, then PEG 8000 (MP Biomedicals) added to a final concentration of 7.5%. The sample was mixed gently but thoroughly for 20 min and then centrifuged at 20,000 *g* for 10 min. The supernatant was brought to a final concentration of 15% PEG 8000 and centrifuged again at 20,000 *g* for 10 min. The pellet was resuspended in 9 ml of buffer A containing 100 µM leupeptin and dialyzed overnight at 4°C in 1 liter of buffer A. The dialyzed sample was applied to a HiTrap DEAE FF Sephacel column (1 ml) (GE Healthcare) previously equilibrated with buffer A. The column was washed with 30 ml of buffer A and the enzyme was eluted with a 15 ml linear NaCl gradient (0±500 mM) in buffer A. Partial purified proteins were stored at −80°C until used for enzyme assay and immunoblotting.

### Enzymatic activity assay of native and recombinant proteins

LKR activity was measured spectrophometrically in the direction of NADPH to NADP^+^ at 25°C. The reaction mixture had a final volume of 1 ml and contained 20 mM _L_-Lysine, 0.1 mM NADPH, 10 mM 2-oxoglutaric acid (neutralized to pH 7.0 with potassium hydroxide), 175 mM Tris-HCl, pH 7.4 and approximately 40 mg of total protein. SDH activity was also measured spectrophotometrically in the direction of NAD^+^ to NADH at 25°C in 1 ml reaction mixture containing 1 mM L-saccharopine, 2 mM NAD^+^ 0.1 M Tris-HCl, (pH 8.5) and approximately 40 mg of total protein.

### LKR/SDH activity staining on a native gel

The partially purified LKR/SDH enzyme from the midgut, ovarian lysates and rGST/LKR, rGST/SDH, His-LKR/SDH recombinant proteins were loaded on 3–10% native gradient polyacrylamide gels (Pagel; ATTO) [10], [25], separated at 20 mA for 120 min and subjected to activity staining. LKR activity staining was performed as follows: the gels were washed in 175 mM Tris/HCl buffer (pH 7.4) for 10 min at 4°C. Following the wash, the gels were incubated for 2 h at 30°C in staining solution containing 20 mM _L_-Lysine, 0.1 mM NADPH, 10 mM 2-oxoglutaric acid (neutralized to pH 7.0 with potassium hydroxide), 0.1% nitro blue tetrazolium and 0.01 mM phenazine methasulfate. The activity band was visualized with UV light as described above. SDH activity was detected as follows: the gels were washed 3 times in 0.1 M Tris buffer (pH 8.5) for 10 min, each wash, at 4°C. Following the wash, the gels were incubated for 2 h at 30°C in staining solution containing 0.1 M Tris HCl buffer (pH 8.5), 1 mM L-saccharopine, 1 mM NAD^+^, 0.1% nitro blue tetrazolium and 0.01 mM phenazine methasulfate. The activity band was visualized with UV light as a bright band against a dark background.

### Immunoblotting analysis

The partially purified LKR/SDH enzyme from the midgut and ovaries, and lysates from eggs, whole tick larva, nymph and adult stages were analyzed by Western blotting using mouse antiserum against rGST/SDH. Tick lysates from eggs, larva, nymph, unfed-tick and fed ticks were prepared as previously described [52]. Eggs, larva, nymphs and adult ticks of *H. longicornis* were homogenized in liquid nitrogen. After homogenization, the lysates were centrifuged at 5000 *g* for 15 min, and the supernatants were stored at −80°C until used for immunoblotting. The tick proteins were separated by SDS-PAGE on a 10% acrylamide gel and 3–10% native gradient polyacrylamide gels (Pagel; ATTO). SDS-PAGE gel was transferred onto a polyvinylidene difluoride (PVDF) membrane (Immobilon-P transfer membrane, Millipore) for immunoblot analysis. The membranes were incubated with the mouse anti-rGST/SDH at a dilution of 1∶100. The binding of antibodies was detected with horseradish peroxidase-conjugated goat anti-mouse IgG (Dako) at a dilution of 1∶1,000 and then placed in substrate solution containing 0.5 mg/ml diaminobenzidine tetrahydrochloride (DAB; Sigma-Aldrich) and 0.005% H_2_O_2_ to visualize the specific antigen bands.

### Indirect fluorescent antibody test (IFAT)

Dissected ovaries and midgut of 4 day post-engorgement ticks were fixed with 4% paraformaldehyde-0.1% glutaraldehyde in PBS overnight at 4°C and embedded in Tissue-Tek O.C.T. Compound (Sakura Finetek). Frozen sections (14 µm) were cut on a cryostat (Leica CM 3050, Leica Microsystems), placed on APS-coated glass slides and then blocked with 5% skim milk in PBS overnight at 4°C. Sections were incubated 30 min at 37°C, with 1∶100 dilution of anti-GST/SDH mouse serum. Anti-GST antibody (1∶100) was used as a negative control. After washing 3 times with PBS, Alexa 488- and Alexa 594-conjugated goat anti-mouse immunoglobulins (1∶1000; Molecular Probes) were applied as second antibodies at 37°C for 30 min. After three washes with PBS, samples were mounted in mounting medium (Vectashield) and then covered with a cover glass, the images were photographed recorded using a fluorescence microscope (Olympus).

### Histochemical detection of SDH activity in sectioned midgut and ovary

Histochemical staining of SDH activity on tick frozen sections was based on the gel staining reaction. Sections were fixed in 0.4% paraformaldehyde at pH 7.0, and 4°C for 2 days, rinsed 4 times in PBS over 12 h at 4°C to remove endogenous substrates, and then incubated for 30 min at room temperature in a reaction mixture containing 4 mM L-saccharopine, 2 mM NAD, 0.1% nitro blue tetrazolium, 0.01 mM phenazine methasulfate and 100 mM Tris-HCl (pH 8.5). Control sections were incubated in the absence of saccharopine. The reactions were stopped by rinsing the sections with double distilled water.

### MitoTracker probes

Sections were fixed in 4% formaldehyde at pH 7.0 and 4°C for 1 h and then rinsed two times with PBS. The fixed cell sections were incubated for 20 min in PBS containing 50 nM of Mitotracker Red CMXRos probe (Invitrogen). After incubation the cell sections were washed 2 times in PBS.

### Starving conditions, and lysine and saccharopine injection protocols

Nymphal ticks were fed on rabbits 3–4 days, then engorged nymphs maintained at 25°C and 70–80% relative humidity chamber until to molt into the adult stage. Molted unfed ticks were collected at 20 days (53), maintained in the 70–80% relative humidity chamber at 15°C for the starved condition. Total RNA was extracted from adult ticks of *H. longicornis* starved for 3 months, 6 months and 10 months to examine the expression levels of mRNA by RT-PCR. For injection experiments, a 50 mM solution of L-lysine (0.5 µl/tick) and 20 mM solution L-saccharopine (0.5 µl/tick) were injected near the fourth coaxes into the hemocoel of unfed adult *H. longicornis* ticks. Control ticks were injected with 0.5 µl PBS. Injected ticks were left for 12 h at 25°C in the incubator to check for mortality resulting from possible injury during injection. Ticks were subsequently fed on the Japanese white rabbit. The ticks were removed after 3 days of blood feeding and total RNA was extracted to determine expression levels of mRNA by RT-PCR. The products of RT-PCR amplification were fractionated by 1.5% agarose gel and the densities of the bands were analyzed by computerized densitometry using Quantity One 1-D Analysis Software (Quantity One Version 4.5, Bio-Rad Laboratories).

### RNA interference of LKR/SDH gene

Templates of partial-length DNA fragments encoding the SDH (588 bp) and LKR domains (680 bp) were amplified by PCR from cDNA clones with the oligonucleotides T7 forward (5′-GGATCCTAATACGACTCACTATAGGCTGGGTCAACAGGACAACCTGCTTACGTCC-3′) and T7 reverse (5′-GGATCCTAATACGACTCACTATAGGTCTACAACGAATTCCCTCGTTCTTGAGC-3′) primers for SDH domain and T7 forward (5′-GGATCCTAATACGACTCACTATAGGAAGAATGGCGTCAAAGTCTA-3′) and T7 reverse (5′-GGATCCTAATACGACTCACTATAGGGTCCTTCGGGTCGACCCAT-3′) primers for LKR to attach the T7 promoter recognition sites (underlined) on both the 5′ and 3′ ends. The PCR products were purified using gel purification kit (Geneclean). The T7 RiboMax^TM^ Express large-scale RNA kit (Promega) was used to synthesize double-stranded RNAs by *in vitro* transcription according to the manufacturer's protocol. Double-stranded RNA/LKR or SDH (2.5 µg/tick) was injected near the fourth coaxes into the hemocoel of unfed adult *H. longicornis* ticks. Control ticks were injected with 0.5 µl PBS alone and firefly luciferase gene (Luc) dsRNA. Injected ticks were left for 18 h at 25°C in an incubator to check for mortality resulting from possible injury during injection. Ticks were simultaneously fed on a Japanese white rabbit as described by Fujisaki [2], and were recovered from the rabbit on the fourth day of feeding. Four days after attachment, a total of 15 ticks were detached from the host for subsequent experiments including five ticks for RNA extraction, five ticks for preparation of tick protein lysate, and five ticks for dissection. Thereafter, ticks were homogenized in TRI reagent and 1x PBS for extraction of whole-tick RNA and lysate antigen preparation for Western blot analysis.

### Statistical Analyses

All statistical analyses and graphihing were done with Microsoft Exel software. Reported results are mean values±SD.

### Accession codes

The complete nucleotide sequences reported in this paper have been deposited in the GenBank database under accession number ***AB464837***.

## Supporting Information

Figure S1 Nucleotide sequence of gene encodining LKR/SDH and deduced aminoacid sequences of H. longicornis. The start codon (ATG) and stop codon (TAG) are indicated in bold letters. The amino acid sequence of the LKR domain and SDH domain of LKR/SDH are underlined. CAAT box and endosperm box (E-box) are boxed.
(0.25 MB PDF)

Figure S2 Alignment of the amino acid sequence with various species of LKR/SDH. The deduced amino acid sequence of H. longicornis LKR/SDH was compared with those of Drosophila melanogaster LKR/SDH (AAF52559), Anopheles gambiae (XP314728), Homo sapiens (CAA07619), Mus musculus (CAA12114) and Oncorhynchus mykiss (AAU95502).
(0.60 MB PDF)
