# Supplementary material for: LKR/SDH Plays Important Roles throughout the Tick Life Cycle Including a Long Starvation Period
Source: PLoS One. 2009 Sep 23;4(9):e7136. doi: 10.1371/journal.pone.0007136 (PMC2745569; doi:10.1371/journal.pone.0007136)
Supplement: Figure S1 — Nucleotide sequence of gene encodining LKR/SDH and deduced aminoacid sequences of H. longicornis. The start codon (ATG) and stop codon (TAG) are indicated in bold letters. The amino acid sequence of the LKR domain and SDH domain of LKR/SDH are underlined. CAAT box and endosperm box (E-box) are boxed. (0.25 MB PDF) [file pone.0007136.s001.pdf]

Figure S1. Nucleotide sequence of gene encodining LKR/SDH and deduced amino acid sequences of *H. longicornis*.

CCGGGGAAACGACGGCGTCGAGCGGGCAAGCCAGCAGGCTTGAAGAGGGCTACGTGCAGCAGCTACTACACGAGGACGGTACCCAGCTACTGACAAGGGCAGAAAAAAGACATCTTCCGG 120
TCACCGCCGGCATTTGAAGATCTGAGGAAGCCTGCCGCAACCGGCAACAGTTTGTGAGTCGTGCGCCAAAGAAGTGTGTTGCTAGACGCTTAGTGGGATCTTCCAGAAGTCCTTGCTAGT 240
AGAAGATTCAGACAAGCGATCATGGTTTCACCGCAGTCCTTCATCTAGTCAAAGGCTAGAACGTGCCTGCTTTGTGTCAGTGTGTTAGTCAACTAAATTAGCTTTAGGAAAGGGTCCAAG 360
AGTCATCGAGTAGTGTTCTTCGAGACAGACTTCCTCAGATCCCGTGGAGCGACTGACCTTTTTCTAAGGCAAGGCTATCAGCAAGGCAGCACGACGGCGGTCTTCAAGCGCTCCGAGTCC 480
TCTCCGGTTAAGTGACGTTGACCTTTGTTGGGTGTGGTGCGGCAGCCCGTCGTCGCCTTGTGCCCTGGTCAGTGGTCACCCGGAAGGGTTATCCGGCGCGACGCCTGAAGAGTGCATGCA 600
GCGGAC**CCAAT**TCGTCGAGGGCGAAGTACGGCGCTGCAACTACTGGTCTGAGCACGAAGCCGTGCACCGCGATAACTGGGCATCGAGCGAATATTCCTGGCTCGTCTGTAAACAGATAAA 720
ATGCTGCATATGTTGAAGAATCACCGTCAGGCCTGCCTGAAGGCCATCCTGTCCCGCTGCTCATCCGAGCTACGACCGGCGAGGCATAAGACCATCGCCATTCGGCGCGAAGATGCGTCG 840
1 M L H M L K N H R Q A C L K A I L S R C S S E L R P A R H K T I A I R R E D A S
CTCTGGGAGCGAAGGGCACCGCTAGCACCACACCACGTCCGGGCGTTGACCAAGAATGGCGTCAAAGTCTACGTCCAGCCTTCCAACAGGAGAGCCTACCCCATACAGGCGTACGTCAAC 960
41 L W E R R A P L A P H H V R A L T K N G V K V Y V Q P S N R R A Y P I Q A Y V N
GCGGGCGGCGAGGTCCGTGAGGACATCAGCGACGTGCCGGTCATCATCGGCGTCAAGCAGGTGCCCATCGACCAGCTGCACCCGAACAAGACGTACGTCTTCTTCTCGCACACCATCAAG 1080
81 A G G E V R E D I S D V P V I I G V K Q V P I D Q L H P N K T Y V F F S H T I K
GCGCAGGAGGCCAACATGCCCATGCTCGACGTCATCCTCGAGCGCAACATCCGGCTCATCGACTACGAGCGCATGTGCGACGCCAACGGCTCGCGAGTCGTGGCTTTCGGCAAGTACGCC 1200
121 A Q E A N M P M L D V I L E R N I R L I D Y E R M C D A N G S R V V A F G K Y A
GGAAAGGCTGGCATGATCAACATCCTGCACGGCCTCGGGCTCCGGCTGCTCGCGCTGGGCCATCACACGCCCTTCATGCACATCGGGCCGGCGCACAACTACCGCAACAGCGGCATGGCC 1320
161 G K A G M I N I L H G L G L R L L A L G H H T P F M H I G P A H N Y R N S G M A
AAGCAAGCCGTGAGGGACGCCGGCTATGAGATCGCGCTCGCCATGATGCCGCGTTCCATCGGACCGCTCACCTTCGTCTTCACCGGATCCGGAAACGTATCCCAGGGTGCGCAAGACATT 1440
201 K Q A V R D A G Y E I A L A M M P R S I G P L T F V F T G S G N V S Q G A Q D I
TTCGAGTCTCTGCCGTGCGAATGGGTCGACCCGAAGGACCTACGTGAAGTTTCAGAGCAGGGCTCCATCACCAAGGTCTACGGTGCCGTGGTCAGCAGGGACGACCACTACCGGCGGATT 1560
241 F E S L P C E W V D P K D L R E V S E Q G S I T K V Y G A V V S R D D H Y R R I
GAGGACGACCACTTCGACCCGGAAGAATGCGACCAGTACCCAGAACGCTACTACTCCACGTTCTCCAAGGACATCGCCCCGTACGCGTCGGTGATAGTGAACGGCATCTATTGGGCGGTG 1680
281 E D D H F D P E E C D Q Y P E R Y Y S T F S K D I A P Y A S V I V N G I Y W A V
AACTCGCCCAAGCTGCTGACCATTCCGGACGCCAAGCGGCTCCTGCAGCCTACCAACACCCCATGGCTGCCGAGCAGTGCGGGGGCGCCGGCGCTGCCCCACCGGCTGCTTGCCATCTGC 1800
321 N S P K L L T I P D A K R L L Q P T N T P W L P S S A G A P A L P H R L L A I C
GACATCAGCGCCGACCCGGGCGGCTCCATCGAGTTCATGAACGAGTGCACCACCATCGATGCACCCTTCTGCCTCTACGACGCTGACCAGCACAAGAACACCGAGAGCTTTGCGGGTCCT 1920
361 D I S A D P G G S I E F M N E C T T I D A P F C L Y D A D Q H K N T E S F A G P
GGCGTTCTTGTCTGCTCCATCGACAACATGCCAACACAGCTTCCCCTAGAAGCCACTGACTACTTCGGCAAGCTGCTCATGCCATACATTGATGACATTATTACGTCCGACGCCACGAAG 2040
401 G V L V C S I D N M P T Q L P L E A T D Y F G K L L M P Y I D D I I T S D A T K
CCGCTGTCTCAGCACCGGATGTCCCCAGTCGTCGAAGGGGCAGTCATCGCCAG**CAAT**GGTAAGCTGACGCCCAACTACGAGTACATCGAAGACTTGAGGAACACGTCAAGGTCTATGAAG 2160
441 P L S Q H R M S P V V E G A V I A S N G K L T P N Y E Y I E D L R N T S R S M K
AAGGCACAGAGCGCGACGGCTGCAAAGATGAAGAAAGTGTTGGTGCTTGGAGCGGGCTACGTGGCCGCACCTCTCGTCGAGTACCTGACGAGGGACAACTCCGTGAATGTCATTGTTGGT 2280
481 K A Q S A T A A K M K K V L V L G A G Y V A A P L V E Y L T R D N S V N V I V G
ACGGCATTCCAGAAGGAAGGCGAGTCTCTAGCCATGAAGTCGCCTAACACGGAGTCCGTGGTCGTGGACGTGATGAAGGCTCCGGACGCCGTGCAGAACCTTGTCAAGGACGCCGATTTA 2400
521 T A F Q K E G E S L A M K S P N T E S V V V D V M K A P D A V Q N L V K D A D L
GTGGTCAGCCTGCTGCCTTATCCGCTTCATCCGACGATTGCCCACCATTGCATCAGGCACGGAATAAACATGGTCACCGCCAGCTACCTCACTTCTGAAATGAAAGAACTGCACGGCGCG 2520
561 V V S L L P Y P L H P T I A H H C I R H G I N M V T A S Y L T S E M K E L H G A
GCTGTCGACGCTAACATCACAGTGCTGAACGAAGTTGGACTTGATCCCGGCATTGACCACCTTCTTGCCATGGAGTGCTTCGACGAAGTGCGGAGGAAGGGCGGCAAGCTCCTCTCGTTC 2640
601 A V D A N I T V L N E V G L D P G I D H L L A M E C F D E V R R K G G K L L S F
GTGTCCTACTGTGGCGGACTGCCAGCGCCAGAGCATGCTAACAACCCACTGCGTTACAAGATCAGCTGGAGCCCACGAAGTGCCTTCATCAACTGTATGGGACCGGCGCGGTATCTAGAA 2760
641 V S Y C G G L P A P E H A N N P L R Y K I S W S P R S A F I N C M G P A R Y L E
AACGACAAGGAAGTGGAGATTCCACCAGGAAGCCTCCTGGACAATGCTCACGAGGTGTCGTTCCTGCCAGGCTTCAACCTAGAGGGGTACCCGAACCGTGACTCCCTCATCTACAAGGCC 2880
681 N D K E V E I P P G S L L D N A H E V S F L P G F N L E G Y P N R D S L I Y K A
ACATATGGAATCAGCAACGCGCATACGGTGCTCCGGGGCACGCTGCGATACAAGGGATTCTCGAGTGCCATGAAAGGGTTGCAGCTCCTAGGACTGCTCGGGGATGAGCCACATCCCAGC 3000
721 T Y G I S N A H T V L R G T L R Y K G F S S A M K G L Q L L G L L G D E P H P S
TTGCATCCCCGGGGACCTGAAATTACCTGGCGCCAGTTTATGACGACACTGCTGGGTCAACAGGACAACCTGCTTACGTCCAACATCAAGAATCTCATCTACGAAAGGGTCGACAAGTGC 3120
761 L H P R G P E I T W R Q F M T T L L G Q Q D N L L T S N I K N L I Y E R V D K C
GAATTGAGGACAAAAGCCATTGAAGATCTGGGTCTCATCGACGACATTCCTGTTGAGAAGAAAAACACTCCCCTGCAGACGCTTATCTTCCACCTTTCCAACAGGCTTGCATACGAACCT 3240
801 E L R T K A I E D L G L I D D I P V E K K N T P L Q T L I F H L S N R L A Y E P
GGTGAGAGGGACCTAGTCATCATGAGACACGACATAGGCATTCAGTGGCACGATGAGAAGAAGGAGGTGAGGCACGTCGACATGGTCACCTACGGCGACC**CCAAT**GGTTACTCGGCCATG 3360
841 G E R D L V I M R H D I G I Q W H D E K K E V R H V D M V T Y G D P N G Y S A M
GCGAAAACCGTCGGCTACCCTGCAGCCATTGCGGCAAAGATGATCTTGCAGGGCGAGATTCAAGCCAAGGGAATGGTGCTGCCGTTTGCTCAGGAAATCTACGGCCCGATGCTCCAGAGG 3480
881 A K T V G Y P A A I A A K M I L Q G E I Q A K G M V L P F A Q E I Y G P M L Q R
CTCAAGAACGAGGGAATTCGTTGTAGAGAAACAACGAGCAAGAACTACTCC**TAG**ACGTGCATTCAACCCTCTTTGCAAAGAGACAACCACTCTTAGGAATCGTACGCTGTGGTGTTATGC 3600
937 L K N E G I R C R E T T S K N Y S *
CTCAGTACCAATGCTACACGCGACGATGGAAAACCGTACCTCAAAATTGGCGTCGCGCCGCAGTGATCTGAATGGGCCTGGACTGCGTCATCGCTGAGCCCAGTCGCCTTCACTGCCGTT 3720
CTTTGTGTACATAAACAATAGCCGTGGCTTTTGTGCCCGGTCAGTGCCGCCATATTCTTAATTTTTTTAAACAAATTATGCAATAAACGTTGCTCTGGTATTTCACAGTACTAAGTTGCA 3840
TTTTCCAGACGAATGTAACATGCAAATCTCTGAATACCTCTCAAGCGGGGCAAAAAATGTATCTACGGGAGCATTATGCTGCTGATGCTGCGAAAAATGTCAAGTTGCAGTTTAAAACTG 3960
GGTTGGTGTTGCAAAGCTAGTAGCGCTTCACGAAGGTGAACACCAGGATGTTTCCTTTCTTATGTCTTTTCTCACTCTGAAGGATTCTTCCTGTCCATCGCAGCCATGTACCTTCTATTT 4080
CAGTAACCAGGTATTCCACATATGCCTTTCAACGACCATTGTTTAGAGCATAAATGATGCAGGGAAGTCACGTGGTGCATGATGGTACGGCGCATGCTGCCAAAGCAACAGGCGTCATTT 4200
GGAGCGGAGGCAAAGACGAGTGATGGACTGCATAAATCTCAGAGCGCGTCATAAAGATCTACCGATGGATCCAATAGAATATCCCTTAGGGTTTCTTTGCAAAAACATGTTGGTGACTAG 4320
GTTTTCGTAGAGTGTATTTGGTCATCCCGGCACCTCAGGTTTCTTACCTTTTATTTTAATTAACGCAGATTTCATGTCACCGCCTTGCATACAAGTGCAGCAAGGTCGGGTACACACTTA 4440
TCTTGTAGCGTATCCACGGGAAATAAAAACTCCTTTACCACGGAAAAAAAAAAAAAAAAAAA 4502
