# Supplementary material for: LKR/SDH Plays Important Roles throughout the Tick Life Cycle Including a Long Starvation Period
Source: PLoS One. 2009 Sep 23;4(9):e7136. doi: 10.1371/journal.pone.0007136 (PMC2745569; doi:10.1371/journal.pone.0007136)
Supplement: Figure S2 — Alignment of the amino acid sequence with various species of LKR/SDH. The deduced amino acid sequence of H. longicornis LKR/SDH was compared with those of Drosophila melanogaster LKR/SDH (AAF52559), Anopheles gambiae (XP314728), Homo sapiens (CAA07619), Mus musculus (CAA12114) and Oncorhynchus mykiss (AAU95502). (0.60 MB PDF) [file pone.0007136.s002.pdf]

Figure S2. Alignment of the amino acid sequence with various species of LKR/SDH.

```
H. longicornis    MLHMLKNHRQACLKAILSRCSSELRPARHKTIAIRREDASLWERRAPLAPHHVRALTKNGVKVYVQPSNRRAYPIQAYVNAGGEVREDISDVPVIIGVKQ 100
D. melanogaster          MWRVIQLRATIAHPFTRQR—HSRVIAIRREDQSVWERRAPFGPTHVQKLVKQNVKVIVQPSNRRAYPMQAYMQAGAHIQEDISDASVIFGVKQ  92
A. gambiae                                     KVIALRREDQSVWERRASFSP-ANVKKLIKQGVKVIVQPSNRRAYMQAYLNAGATVQEDISEASVIFGVKQ  71
H. sapiens           MLQVHRTGLGRLGVSLS---KGLHHKAVLAVRREDVNAWERRAPLAPKHIKGITNLGYKVLIQPSNRRAIHDKDYVKAGGILQEDISEACLILGVKR  94
M. musculus          MLRAQRPRLARLRACLS---RGLHHKPVMALRREDVNAWERRAPLAPKHIKGITKLGYKVLIQPSNRRAIHDKEYVRAGGILQEDITEACLILGVKR  94
O. mykiss            MLRLLRHQARGTRSCLCGQRRYEHHKSVMAIRREDINVWERRAPLAPRHVKEIVHAGHKVLVQPSNRRAIHENYYEKAGAIISEDISEASLIIGVKS  97

H. longicornis    VPIDQLHPNKTYVFFSHTIKAQEANMPMLDVILERNIRLIDYERMCDANGSRVVAFGKYAGKAGMINILHGLGLRLLALGHHTPFMHIGPAHNYRNSGMA  200
D. melanogaster   VPIDALIPGKTYCFFSHTIKAQESNMPLLDAILEKKIRLIDYERIIDERGARQVAFGKYAGVAGMVNILHGIGLRLLALGHHTPFMHIGPAHNYRNSSMA  192
A. gambiae        VPVDALIPQKTYCFFSHTIKAQESNMPLLDACLEKNIRLIDYEKLMDRNGQRLVAFGKYAGVAGMVNILHGLGLRLLALGHHTPFMHVGPAHNYRNSSMA  171
H. sapiens        PPEEKLMSRKTYAFFSHTIKAQEANMGLLDEILKQEIRLIDYEKMVDHRGVRVVAFGQWAGVAGMINILHGMGLRLLALGHHTPFMHIGMAHNYRNSSQA  194
M. musculus       PPEEKLMSKKTYAFFSHTIKAQEANMNLLDEVLKQEIRLIDYEKMVDHRGSRIVAFGQWAGVAGMINILHGMGLRLLALGHHTPFMHLGMAHNYRNSSQA  194
O. mykiss         PPEEKLYPRKTYAFFSHTIKAQEANMGLLDDLLKKEVRLIDYEKMVDANGFRIVAFGQWAGVAGMINILHGLGLRFLALGHHTPFMHIGMAHNYRNVSQA  197

H. longicornis    KQAVRDAGYEIALAMMPRSIGPLTFVFTGSGNVSQGAQDIFESLPCEWVDPKDLREVSEQGSITKVYGAVVSRDDHYRRIEDDHFDPEECDQYPERYYST  300
D. melanogaster   RQAIRDCGYEISLGMMPKSIGPLTFVFTGSGNVSQGAQEVFSELPIEYVPPEMLRKVAEHGNQNKLYGCEVSRSDHLERREGGGFDAKEYDEFPERYIST  292
A. gambiae        RQAVRDCGYEISLGMMPKSIGPLTFIFTGSGNVSQGAQEVFQELPVEFVPPEMLRKVAEHGSTNKLYGCEVSRSDHLERREGGKFDPVEYDQYPERYVST  271
H. sapiens        VQAVRDAGYEISLGLMPKSIGPLTFVFTGTGNVSKGAQAIFNELPCEYVEPHELKEVSQTGDLRKVYGTVLSRHHHLVRKTDAVYDPAEYDKHPERYISR  294
M. musculus       VQAIRDAGYEISLGLMPKSIGPLTFVFTGTGNVSKGAQEVFNELPCEYVEPHELREVSKTGDLRKVYGTVLSRHHHLVRKTDGVYDPVEYEKYPERYTSR  294
O. mykiss         IQAVRDCGYEISMGLMPKSIGPVTFVFTGTGNVSKGAQDIINELPVEYVEPHELKDVSQTGDMSRVYATVLSRHHHLMRKSDGVYDPLEYEYHPELYTSH  297

H. longicornis    FSKDIAPYASVIVNGIYWAVNSPKLLTIPDAKRLLQPTNTPWLPSSAGAPALPHRLLAICDISADPGGSIEFMNECTTIDAPFCLYDADQHKNTESFAGP  400
D. melanogaster   FSTKIAPYASVIVNGIYWAVGSPKLISIPDAKNLLRPANTPWLPTSKGSPALPHRMLAICDISADPGGSIEFMNECTTIDTPFCLYDADRNKDTKSFKGP  392
A. gambiae        FSKNIAPYASVIVNGIYWAVGAPKLITIPDAKNLLRPANTPWLPTSRGSPALPHRMLAICDISADPGGSIEFMNECTTIDTPFCLYDADRNKDQKSFKGP  371
H. sapiens        FNTDIAPYTTCLINGIYWEQNTPRLLTRQDAQSLLAPGKFSPAGV-EGCPALPHKLVAICDISADTGGSIEFMTECTTIEHPFCMYDADQHIIHDSVEGS  393
M. musculus       FNTDIAPYTTCLINGIYWEQNTPRLLTRQDAQSLLVPVKSSVVPV-EGCPELPHKLVAICDISADTGGSIDFMTECTTIERPFCMYDADQQIIHDSVEGS  393
O. mykiss         FRTSVAPYTTCLINGIYWDPQTPRLLRRLDAQRLLTHVKPSAAAT-EGWPELPHKLLAICDISADMGGSIEFMTECTSIDKPFCMYDADQHIDHDSVEGT  396

H. longicornis    GVLVCSIDNMPTQLPLEATDYFGKLLMPYIDDIITSDATKPLSQHRMSPVVEGAVIASNGKLTPNYEYIEDLRNTS--RSMKKAQSATAAKMKKVLVLGA  498
D. melanogaster   GVLVCSIDNMPTQLPRESTDLFGELLAPHVHDIIKSDAKKPLAEENFSYPIQSAIIASNGQLTEGFQYIQELRESQSHRSRHKMEGSSESD-KKVLVLGA  491
A. gambiae        GVLVCSIDNMPTQLPREATDFFGDLLYPYALDILQSDASRPLEEHNFCQPIQSAIIASNGQLTEGFQYIQELRESQSHRSRHKMEGSSESD-KKVLVLGA  491
H. sapiens        GILMCSIDNLPAQLPIEATECFGDMLYPYVEEMILSDATQPLESQNFSPVVEGAIICSNGNLTPGFEYINELREMN-NRSRHKTEGSYEGK-KRVLVLGA  469
M. musculus       GILMCSIDNLPAQLPIEATEYFGDMLYPYVEEMLLSDASQPLESQNFSPVVRDAVITSNGTLPDKYKYIQTLRESR-----ERAQSLSMGTRRKVLVLGS  488
O. mykiss         GILMCSIDNLPAQLPIEATEYFGDRLFPYIWEMLLSDATRPLEEEDFSPQVRDAVITSEGKLTPKFEYIEDLRQRS-----EQAKIMKRSGMKRVLLLGS  491

H. longicornis    GYVAAPLVEYLTRDNSVNVIVGTAFQKEGESLAMKSPNTESVVVDVMKAPDAVQNLVKDADLVVSLLPYPLHPTIAHHCIRHGINMVTASYLTSEMKELH  598
D. melanogaster   GMVSAPLVEWLHREKDVSITVCSQVKEEADRLAQQYAGVDSVYLDVNESTGHLQELCGRADVVVSLLPYSLHGMVARYCVAEGTHMVTASYLNDEISGLH  591
A. gambiae        GFVSAPLVEYLHRESNVSIKVASQYKEEADRLAHRYQGVESVYVNVQDESANLQNLCEESDVVVSLLPYSLHSVIAKHCIAGKTHLVTASYVNDDISALH  569
H. sapiens        GYISEPVLEYLSRDGNIEITVGSDMKNQIEQLGKKY-NINPVSMDICKQEEKLGFLVAKQDLVISLLPYVLHPLVAKACITNKVNMVTASYITPALKELE  587
M. musculus       GYVSGPVLEYLSRDNNIEITLGSDMTNQMQQLSKKY-NINPVSLTVGKQEAKLQSLVESQDLVISLLPYVLHPVVAKACIESRVNMVTASYITPAMKELE  587
O. mykiss         GYVSGPVIEYLTRDPGTQITVASVLLTQAEELAGKYPNTIPVMLDVTSQEGHLESLVKDHDLVISMLPYGYHPVIAKHCINKKVNMVTASYLSPAMKDLQ  591

H. longicornis    GAAVDANITVLNEVGLDPGIDHLLAMECFDEVRRKGGKLLSFVSYCGGLPAPEHANNPLRYKISWSPRSAFINCMGPARYLENDKEVEIPPGS-LLDNAH  697
D. melanogaster   EEAKAKGVTIMNEVGLDPGIDHLLALECIHEVQDKGAVVESFVSYCGGLPAPEHSNNALRYKFSWSPRGVLLNTLSAAKYLSQGQIVEISGGGELMSSPR  691
A. gambiae        SAAQDAGVTIMNEVGLDPGIDHLLALECIKDVQENGGVVESFVSFCGGLPAPEHSNNPLRYKFSWSPRGVLLNTLSAAKYLSKGQVVEISGGGELMSAPR  669
H. sapiens        KCVEDAGITIIGELGLDPGLDHMLAMETIDKAKEVGATIESYISYCGGLPAPEHSNNPLRYKFSWSPVGVLMNVMQSATYLLDGKVVNVAGGISFLDAVT  687
M. musculus       KSVDDAGITVIGELGLDPGLDHMLAMETIDTAKELGATVESYVSYCGGLPAPEHSDNPLRYKFSWSPVGVLMNIMQPASYLLNGKVVNVTGGVSFLNSVT  687
O. mykiss         QSAEEAGITIVNEMGLDPGIDHMLAMECIDQAKADGCTIESYSSFCGGLPAPECSDNPLRYKFSWSPYGVLLNTISPAIFLKDNEVVSIPAGGTLMESTS  691

H. longicornis    EVSFLPGFNLEGYPNRDSLIYKATYGIS-NAHTVLRGTLRYKGFSSAMKGLQLLGLLGDEPHPSLHPRGPEITWRQFMTTLLGQQD-NLLTSNIKNLIYE  795
D. melanogaster   SLDFLPGFALEGFPNRDSTKYGNLYGLGRDVHTLLRGTIRYKGFSESIKPMQLLGLIDPEPHALLHPSGPDVTWRQLVIHLMGMSDSTIFYENLKQKLTE  791
A. gambiae        ELEFLPGFALEGFPNRDSTKYQSLYGLT-NINTLLRGTIRYKGFSDTIKPMQLLGLIDPNPHPLLHPHGPELTWRQLVVNMLGLADADIFIENLKHRLAE  768
H. sapiens        SMDFFPGLNLEGYPNRDSTKYAEIYGIS-SAHTLLRGTLRYKGYMKALNGFVKLGLINREALPAFRPEANPLTWKQLLCDLVGISP-SSEHDVLKEAVLK  785
M. musculus       PMDYFPGLNLEGYPNRDSIKYAEIYGIS-SAHTLLRGTLRYKGYSKALNGFVKLGLINREAYPALRPEANPLTWKQLLCDLVGISR-SSPCEKLKEVVFT  785
O. mykiss         PMDFLPGFNLEGFPNRDSTKYSEQYGIE-SAHTLIRGTLRFKGFSKAMSGFVKLGLINTDPCPMLKHTSAPVSWKELLCNQIGLHP-STSDKAFEGAVYD  789

H. longicornis    RVDKCELRTKAIEDLGLIDDIPVEKKNTPLQTLIFHLSNRLAYEPGERDLVIMRHDIGIQWHDEKKEVRHVDMVTYGDPN---GYSAMAKTVGYPAAIAA  892
D. melanogaster   RIGDVDG----IESLGLLDDTPVVKLNTPLDTLSHYLSKRLAFERDERDLVVLRHEVGIRWPDGRREERGINFVVYGQPQ---GHSAMAMTVGKPAAIAA  884
A. gambiae        RVGTIEG----LEELGLLDNVPVVKMGSPLDTLSYYLSKKLAFEDTERDLIILRHDVGIRWSDGRREERGINFVVYGQPASTGGHSAMAKTVGFPAAIAA  864
H. sapiens        KLGGDNTQLEAAEWLGLLGDEQVPQAESILDALSKHLVMKLSYGPEEKDMIVMRDSFGIRHPSGHLEHKTIDLVAYGDIN---GFSAMAKTVGLPTAMAA  882
M. musculus       KLGGDNTQLEAAEWLGLLGDEQVPQAESIVDAFSKHLVSKLSYGPEEKDMIVMRDSFGIRHPSGHAENKTIDLVVYGDFN---GFSAMAKTVGLPTAMAA  882
O. mykiss         RIGQDPFKMETLKWFGMLSKEAVPHAETVLASLAKHLEARLSFDEGERDMIIMRNDVGLRHSTGELETKHISLVVYGDSN---GFSAMAKTVGYPAAIAA  886

H. longicornis    KMILQGEIQAKGMVLPFAQEIYGPMLQRLKNEGIRCRETTSKNYS  937        Id.        Sm.
D. melanogaster   KMILDGEIQERGVLLPFTPDIYRPMLQRLRSEGLTATETSRWLN   928        61%        15%
A. gambiae        KMIIDGEIQQRGVVLPFSADIYRPMLARLEQEGLTATTTTKVL    907        62%        15%
H. sapiens        KMLLDGEIGAKGLMGPFSKEIYGPILERIKAEGIIYTTQSTIKP   926        53%        17%
M. musculus       KMLLDGEIEAKGLMGPFTKEIYGPILERIKAEGIVFNTQSTIKL   926        53%        18%
O. mykiss         RMVLDGEIRTKGLVVPMTKDIYGPALKRLQEEGLKFTSKSTIQE   930        52%        19%
```
